# Development and Evaluation of an e-Book for Bone Health and Osteoporosis Education in Adolescents

**DOI:** 10.3390/nu15081899

**Published:** 2023-04-14

**Authors:** Nur Filzah Insyirah Mohd Azmi, Muhammad Hafiz Aznul Hisham, Nor Aini Jamil

**Affiliations:** Centre for Community Health Studies (ReaCH), Faculty of Health Sciences, Universiti Kebangsaan Malaysia, Kuala Lumpur 50300, Malaysia

**Keywords:** bone health, adolescents, education, osteoporosis, e-book

## Abstract

Achieving optimum peak bone mass during adolescence is crucial for lifetime bone health. This study is aimed at developing and assessing an e-book designed for adolescent bone health knowledge and osteoporosis education. A needs assessment was conducted among 43 adolescents, aged 13–16 years, living in urban areas in Malaysia to determine their needs and preference for health educational material. The researchers also searched for relevant guidelines and articles regarding adolescent bone health. Subsequently, an e-book was developed based on the needs assessment and literature search. Five expert panelists (mean work experience = 11.3 years) validated the e-book contents and assessed its understandability and actionability using the Patient Educational Materials Assessment Tool for Audio-Visual Material (PEMAT-A/V). The internet (72.1%), parents (44.2%), television (41.9%), and teachers (39.5%) were the top four sources of health information among the respondents. Magazines (4.6%) and newspapers (11.6%) were the least preferred resources. Most adolescents were interested in cartoon-themed educational materials, and they perceived that including a short video, quiz, and infographic in the educational material would make it much more interactive for users. The developed e-book includes seven infographic chapters, a link to a quiz, and a summary video. The topics cover basic information on bones and the formation and resorption processes, osteoporosis and its risk factors, key nutrients for bone health (calcium and vitamin D), along with their sources and recommended amounts, physical activity and exercise for bone health, and tips for healthy lifestyle practices for bone health. All chapters and the video were rated with a median score of 100% for understandability and actionability, respectively. Some of the comments received from evaluators included that the e-book made good use of infographics, was easy to understand and interesting, and contained well-organized content. Some suggestions for improvement included adding take-home messages relevant to the topic, using colors to highlight keywords, and narrating all points presented in the video. Overall, the newly developed e-book on adolescent bone health was highly rated by expert panelists. However, the acceptance and effectiveness of the e-book in increasing bone health and osteoporosis knowledge among adolescents are yet to be evaluated. The e-book could be used as one of the educational tools to promote bone health in adolescents.

## 1. Introduction

Bone is a dynamic connective tissue that constantly changes its shape and mass in response to the environment [1,2]. The bone matrix becomes mineralized with calcium and phosphate deposits, which give strength to the structure. Notable changes in bone size, mass, and structure occur in children and adolescents during a growth spurt when the rate of bone formation is greater than the rate of bone resorption [3]. Before puberty, there is no significant difference in bone mineral density between boys and girls. Peak bone mass (PBM), defined as the maximum amount of bone mass an individual can attain, is achieved during late adolescence between the ages of 18 and 25. Girls reach PBM earlier than boys, resulting in a greater bone mass content in boys due to a more extended period of bone growth than that experienced by girls. Following PBM, bone mass is maintained throughout adulthood and gradually declines around the age of 40 [4]. However, bone loss is more significant in women following menopause due to the decline in the secretion of estrogen [5].

Osteoporosis is the most common metabolic bone disease in older adults and is known as a ‘silent disease’ because there are no symptoms until a bone fracture occurs [6]. Osteoporosis is characterized by a decrease in bone mineral density, strength, and quality, resulting in thin, weak, and fragile bones that could break easily. Globally, one in three women and one in five men aged 50 and above will develop osteoporosis [7]. Osteoporosis occurs when bone density decreases due to a faster rate of bone resorption than the rate of bone formation. Aging, genetics, and lifestyle choices are all fundamental causes of osteoporosis [8]. In Asian countries, hip fractures are projected to double by 2050 compared to 2018 [9].

There are a number of modifiable risk factors associated with osteoporosis prevention, including healthy dietary practices and regular physical activity [10]. A healthy bone diet should emphasize an adequate amount of calcium intake from food for bone mineralization [7]. In general, calcium recommendations vary by age group, with children and adolescents having a high requirement to support rapid bone growth and attainment of PBM [11]. Milk and dairy products are good sources of calcium, and it has been shown that individuals who do not consume dairy products during childhood are more likely to develop fragility fractures during adulthood [12]. Another important nutrient for bone health is vitamin D, which helps with dietary calcium absorption. Vitamin D can be obtained through sun exposure as well as diet, but foods naturally containing vitamin D (e.g., salmon, sardines, tuna, and egg yolks) are limited and not widely consumed. Rickets in children is caused by a lack of vitamin D, characterized by thin, fragile, and poorly formed bones.

Aside from that, a healthy lifestyle that includes weight-bearing exercises on a regular basis is essential for increasing bone density and stimulating the formation of new bones [8,13]. In general, mechanical forces applied to the bone promote bone formation, while weight-bearing exercises improve bone mineral deposition. In addition, high-impact physical activities, such as jumping, also help in the accumulation of bone minerals in children and adolescents. Furthermore, some evidence suggests that exercise-induced bone mass gains in children are maintained into adulthood, implying that physical activity habits developed in childhood may have long-term benefits for bone health [14]. Meanwhile, smoking and excessive alcohol intake are risk factors for osteoporosis and should be avoided [15,16].

It has been shown that a person with optimal PBM is less likely to develop osteoporosis and fragility fractures later in life, as they possess a greater ‘bank’ of calcium in the bone. The greater the calcium reserves in the bone, the lower the risk of developing osteoporosis [17]. A 10% increase in PBM will result in a 50% reduction in the risk of fracture in older adults [18]. Since everyone loses bone mass as they age, achieving optimal PBM during adolescence is critical for maintaining good bone health in the long term [19]. Osteoporotic fractures may lead to substantial disability, in addition to an increased risk of morbidity and mortality [20].

Although sufficient intake of calcium is necessary for the healthy development of bones during childhood, adolescents are at risk of poor dietary calcium consumption [21,22]. Meanwhile, the high prevalence of vitamin D deficiency among children and adolescents worldwide is associated with reduced sunlight exposure and poor dietary habits [23,24]. Furthermore, a global study of 1.6 million school-aged adolescents, 11–17 years old, found that most adolescents do not meet the current daily physical activity recommendations due to changes in physical activity behavior [25]. These concerning findings are supported by a study that discovered that most adolescents and young adults from various nations have low knowledge of osteoporosis, resulting in poor osteoporosis prevention measures [26].

Therefore, education on osteoporosis prevention is vital in the early years, as adolescence is when biological, psychosocial, cognitive, and emotional changes occur, making individuals more vulnerable to engaging in risky behaviors [27,28]. Educational intervention is one of the most effective strategies for improving bone health in teenagers by increasing bone health knowledge, attitude, and dietary practice [29]. A study reported that adolescents prefer online resources that include concise messages that are easily accessible and contain actionable information that can be implemented in daily life [30]. Illustrations, pictures, animation, hyperlinks, sound, and video in educational material provide interactive aspects [31].

All of the above mentioned characteristics are included in the e-book format, which may facilitate more enjoyable learning for its readers [32]. E-books are user-friendly and flexible electronic multimedia that can digitally include features such as sound, video, animation, graphics, and text [33]. Furthermore, they are easily accessible online using handheld devices in various file and reader formats [32]. The aim of this study is to develop and evaluate an e-book for bone health and osteoporosis education as an educational material for teenagers.

## 2. Materials and Methods

### 2.1. Study Design

This study consisted of three phases: (i) a needs assessment regarding the characteristics and preference for health educational material among adolescents, (ii) the development of the bone health and osteoporosis educational material, and (iii) an expert panel evaluation of the educational material. The University’s Research Ethics Committee (reference code: UKM PPI/111/8/JEP-2022-139) approved the study. All study participants provided their written informed consent prior to participation in the study.

### 2.2. Phase 1: A Needs Assessment of Health Education Characteristics and Preferences among Adolescents

An online survey was conducted using Google Forms to determine the preferred characteristics of health educational material and information sources among healthy Malaysian urban adolescents, aged 13–16. We confirmed the urban location by using their postcode. Adolescents with hearing impairments, as well as physical and mental disabilities, were excluded. Apart from that, a literature search was performed using electronic databases, including PubMed and Google Scholar, to identify relevant guidelines for adolescent bone health. Websites of established organizations, such as the International Osteoporosis Foundation (IOF) and the Bone Health and the Osteoporosis Foundation, were also referenced as adolescent bone health educational materials. All information was extracted and summarized in a table to guide the contents and sub-topics of the e-book.

### 2.3. Phase 2: Development of the Adolescents’ Bone Health and Osteoporosis Educational Material

Based on the needs assessments and literature search conducted earlier, an e-book for bone health and osteoporosis education for teenagers was developed. The e-book was written in Malay and designed to include infographics using Canva (Canva Pty Ltd., Australia). In addition, a video was created using Powtoon (Powtoon Ltd., UK) to summarize the key points and tips for healthy lifestyle practices for good bone health. Meanwhile, a short quiz was created on Quizizz, a game-based learning platform, to assess readers’ understanding and to add a fun factor. Readers could access the video and quiz via QR codes attached to the e-book. Finally, the e-book was uploaded online using Paperturn.com, an online flipbook software.

### 2.4. Expert Panel Evaluation of the Adolescents’ Bone Health and Osteoporosis Educational Material

A panel of nutrition/dietetics experts and active bone health researchers from a medical university in Kuala Lumpur, Malaysia, with at least three years of experience, were invited to evaluate the educational material. We excluded those with non-Malaysia nationality. The number of expert panels recommended by the previous study was five [34]. Firstly, the researchers held a briefing session with expert panels via Microsoft Teams to introduce the educational material and evaluation procedures. Subsequently, each panel received a link to a Google Drive containing: (i) an overview of the e-book and evaluation procedures and (ii) folders containing the e-book sub-topic in PDF format, along with a video with its evaluation form.

The educational material was evaluated using the Malay version of the Patient Education Materials Assessment Tool for Audio-Visual Material (PEMAT A/V) [35,36]. This instrument evaluates two domains: understandability (13 items) and actionability (4 items). Understandability is a user’s ability to understand and communicate key information in educational material. In contrast, actionability is defined as a user’s ability to put provided information into action, regardless of background or health literacy level [35]. Each item scored either 1 (agree), 0 (disagree), or N/A (not applicable). Each domain’s total score was computed and presented as a percentage (%). The higher the score, the easier the material is to understand or put into practice. At the end of the evaluation form, there is a space for panels to provide comments and suggestions for each chapter and video. The panels had two weeks to finish the independent evaluation process.

## 3. Results

### 3.1. Phase 1: A Needs Assessment of Health Education Characteristics and Preferences among Adolescents

A total of 43 adolescents (mean age 16.6 years) took part in the survey (Table 1). The majority of the respondents were females (65%), Malay (98%), and from the southern region of Malaysia (72%).

Table 2 shows the source of reference for health information among adolescents. The top four resources were the internet (72.1%), parents (44.2%), television (41.9%), and teachers (39.5%). Conversely, magazines (4.6%) and newspapers (11.6%) were the respondents’ least preferred sources of health information.

For the preferred characteristics of health education materials, most of the respondents were interested in educational materials in Malay with a cartoon theme (Table 3). The respondents also perceived that incorporating a short video, quiz, and infographic into the educational material would make it much more interactive for users.

### 3.2. Phase 2: Development of the Adolescents’ Bone Health and Osteoporosis Educational Material

Based on the needs assessment and information gathered from the literature search, an e-book for bone health and osteoporosis education was created. The e-book is written in Malay and features vibrant infographics and illustrations, as well as a flip sound and music background. There are 58 pages in total, divided into seven sub-topics, each with less than ten pages (Table 4).

The first chapter focuses on basic bone knowledge, such as bone function, formation, and resorption, as well as the stages of bone growth and loss in the human lifecycle. This is followed by an introduction to osteoporosis, risk factors, and how to diagnose osteoporosis in Chapter 2. In Chapter 3, a special section focusing on the important aspects of lifestyle practices affecting adolescents’ bone health, such as smoking, caffeine, alcohol intake, and eating disorders, is presented. Chapters 4 and 5 discuss calcium and vitamin D as important nutrients for bone health, including their sources, recommended amounts, the total amount per serving size, along with tips for increasing intake. The importance of physical activity and exercise for bone health, including the types, duration, and examples, are covered in Chapter 6. In the final chapter, a summary of overall bone health lifestyle practices that adolescents can implement is provided.

At the end of the e-book, a 5 min video was presented, which can be accessed by scanning the QR code. The video summarizes all of the important aspects of bone health and osteoporosis education, as well as practical tips for adolescents. Finally, to include fun and interactive elements, a pop quiz with ten questions, accessible via QR code, was also added.

### 3.3. Phase 3: Expert Panel Evaluation of the Adolescents’ Bone Health and Osteoporosis Educational Material

Eleven expert panelists were invited, and six agreed to participate in this study. However, one panelist had to withdraw due to work commitments. As a result, five panelists (mean age = 36.5 years) completed the evaluation process of the e-book. Table 5 shows the socio-demographic profiles of the expert panelists. The majority of the panelists were female (80%), Malay (60%), and had backgrounds in nutrition and dietetics (80%). The average work experience of the expert panelists was 11.3 years. In addition, most of the expert panelists (60%) had Doctor of Philosophy (PhD) degrees.

The overall median score for understandability was 100%, ranging from 83% to 100% (Table 6). Five chapters were scored as 100% by all expert panelists. For actionability, the median score was 100%, ranging from 0% to 100%. Four e-book chapters and the video were rated as 100% actionable. Some of the comments received included that the e-book made good use of infographics and included content that was easy to understand, interesting, and well-organized. The panel suggested adding a take-home message for Chapter 1 and incorporating relevant take-home messages for Chapters 2 and 3 to improve the actionability domain. They also suggested using colors to highlight keywords and narrating all points presented in the video at a moderate speed to improve the e-book and the video’s understandability.

## 4. Discussion

The current study successfully developed an e-book for bone health and osteoporosis education based on preferred characteristics among adolescents. The contents of this e-book were evaluated by expert panelists, with a median score of 100% for understandability and actionability. This e-book can potentially educate adolescents about bone health and could act as one of the strategies for the early prevention of osteoporosis.

The needs assessment carried out in the current study highlighted that the internet is the primary source of health information for the study participants. Online health education is not only easily accessible, but it also provides users with free access to a diverse source of health information. Effective online health education material may boost user motivation and adherence to behavioral change toward self-involvement. However, accurate information is critical in developing health educational material to avoid misleading information [37]. Using e-books as online educational material could assist in the delivery of information interactively in multiple media formats, including text visualization, video, music, illustrations, and animations. Compared to the printed book, an e-book has the advantage of being able to be read simultaneously by a large number of people in their preferred comfortable environment [31,38]. E-books are also great learning tools, since they increase learners’ interest, motivation, and stimulation in regards to learning activities [31].

Previous studies reported that magazines and newspapers are the least preferred sources of health information among adolescents, in agreement with our findings [39]. In their study, about 20% of teenagers prefer to read magazines and newspapers, while more than 80% use social media to read information daily. Due to the widespread outbreak of COVID-19, most adolescents have shifted to online learning, which has become their new learning environment [40]. Nevertheless, parents and teachers should continue to play an active role as teenagers’ primary health instructors and should be equipped with appropriate health information in order to encourage adolescents to adopt healthy behaviors by acting as role models or providing verbal reinforcement [41,42].

Our findings also revealed that most adolescents preferred health educational material in their native language and featuring cartoon characters. Educational material written in the native language of the readers will remove language barriers and allow for clear communication in delivering information [43]. Meanwhile, presenting ideas as cartoon illustrations, both pictorially and verbally, stimulates the cognitive development of learners by grabbing their attention and interest. Cartoons can also translate scientific information into a visual language that students can understand [44].

The newly developed e-book on bone health and osteoporosis education created in the current study was highly understandable and actionable among the expert panelists. Users with various health literacy levels would benefit from the educational material that is easily understood and actionable [45]. This e-book, presented in an infographic form, provides fundamental knowledge about bones and osteoporosis, as well as lifestyle practices to promote strong bones. Such information is essential for educating the younger generation, since childhood and adolescence is a critical period for optimum bone mineralization in both men and women. A summary video is provided at the end of the e-book to re-emphasize the information given. Meanwhile, a short quiz is included to increase the interactive element and act as a fun factor.

It is hoped that this educational material will be useful in raising awareness among children and adolescents about the importance of maintaining good bone health. This kind of effort is essential as a preventive measure to lower their risk of developing osteoporosis later in life. However, our review of the relevant literature revealed that there is a dearth of evaluations of educational resources on bone health. In addition, few studies used PEMAT A/V as an evaluation tool for health education materials [46,47].

The present study was the first in Malaysia to develop an e-book for bone health and osteoporosis education for adolescents. Our e-book was created based on a needs assessments conducted among adolescents, and its contents, understandability, and actionability were reviewed and evaluated by expert panelists. This e-book, however, has yet to be evaluated by teenagers. This is necessary to ensure that the target user accepts and benefits from the educational material. Platforms such as online education and leisure services can be used to disseminate this e-book, once its effectiveness and acceptance have been proven. Schools can also promote this e-book to the younger generation and incorporate it into their curriculum.

## 5. Conclusions

An e-book was developed based on a needs assessment of the target group to educate adolescents about bone health and osteoporosis. The content of this e-book was validated as highly understandable and actionable by expert panelists. Future studies should assess the effectiveness of this e-book in increasing bone health knowledge and lifestyle practices among urban adolescents, as well as their acceptance of this educational material. Nevertheless, this e-book could be used as one of the educational tools and reading materials to promote bone health in adolescents.

## Figures and Tables

**Table 1:** Sociodemographic profiles of adolescents in needs assessments (*n* = 43).

Parameter	*n* (%)	Mean (SD)
Age (years)		16.6 (1.5)
Gender		
Male	15 (34.9)	
Female	28 (65.1)	
Ethnicity		
Malay	42 (98.0)	
Chinese	1 (2.0)	
Region		
Southern	31 (72.1)	
Central	12 (27.9)	

**Table 2:** Source of reference for health information among adolescents in needs assessments (*n* = 43).

Source	Frequently/Always	Sometimes	Never
	*n* (%)	*n* (%)	*n* (%)
Internet	31 (72.1)	12 (27.9)	0
Parents	19 (44.2)	24 (55.8)	0
Television	18 (41.9)	23 (53.5)	2 (4.6)
Teachers	17 (39.5)	24 (55.8)	2 (4.6)
Peers	16 (37.2)	26 (60.5)	1 (2.3)
Doctor/Healthcare Professionals	13 (30.2)	25 (58.1)	5 (11.6)
Posters	6 (14.0)	27 (62.8)	10 (23.3)
Newspapers	5 (11.6)	19 (44.2)	19 (44.2)
Magazines	2 (4.6)	17 (39.5)	24 (55.8)

**Table 3:** Preferred characteristics of health education materials among adolescents in needs assessment (*n* = 43).

Domain	Preferred	Less Preferred	Not Interested
	*n* (%)	*n* (%)	*n* (%)
Language			
Malay	41 (95.3)	2 (4.6)	0
English	28 (65.1)	15 (34.9)	0
Theme, design and illustration			
Cartoon	34 (79.1)	9 (20.9)	0
Realism	26 (60.5)	14 (32.6)	3 (7.0)
Minimalist	24 (55.8)	13 (30.2)	6 (14.0)
Interactive activity			
Short video	38 (88.4)	4 (9.3)	1 (2.3)
Quiz	32 (74.4)	9 (20.9)	2 (4.7)
Infographic	30 (69.8)	12 (27.9)	1 (2.3)
Table/chart	21 (48.8)	20 (46.5)	2 (4.7)

**Table 4:** Summary of the e-book contents for adolescent bone health and osteoporosis educational material.

Chapter	Topic	No. of Pages/Video Duration	Contents
1	Let’s know our bones!	9	Introduction to bones Bone formation process Bone function Phases of bone growth and loss
2	Osteoporosis	8	Introduction to osteoporosis Osteoporosis risk factors Diagnosis of osteoporosis Take-home messages
3	Adolescent bone health	9	Importance of bone health in adolescence Adolescent bone health and lifestyle factors Take-home messages
4	Calcium	10	Importance of calcium Calcium intake among Malaysians Recommendation of daily calcium intake Calcium food sources and content per serving Tips to increase daily calcium intake Take-home messages
5	Vitamin D	9	Importance of vitamin D Sources of vitamin D Recommendation of daily vitamin D intake Vitamin D food sources and content per serving Tips for sunlight exposure Take-home messages
6	Physical activity and exercise for bone strength	6	Importance of physical activity and exercise Recommendation of physical activity and exercise Example of weight-bearing and non-weight-bearing exercises Let’s try this muscle-strengthening exercise! Take-home messages
7	Seven healthy lifestyles for bone health	4	Examples of a healthy lifestyle Take-home messages
	Video	5 min	Summary of adolescent bone health and prevention of osteoporosis
	Quiz	-	Consisting of 10 questions (true/false and multiple choice)

**Table 5:** Sociodemographic profiles of expert panels (*n* = 5).

Parameter	*n* (%)	Mean (SD)
Age (years)		36.5 (6.9)
Gender		
Male	1 (20.0)	
Female	4 (80.0)	
Ethnicity		
Malay	3 (60.0)	
Chinese	2 (40.0)	
Profession		
Dietitian	2 (40.0)	
Bone health active researcher	1 (20.0)	
Nutrition/Dietetics lecturer	2 (40.0)	
Work experience (years)		11.3 (5.7)
Education Level		
Degree	1 (20.0)	
Master	1 (20.0)	
PhD	3 (60.0)	

**Table 6:** Scores of understandability and actionability of the e-book for adolescent bone health and osteoporosis educational material.

Chapter	Topic	Understandability Score (%)			Actionability Score (%)		
		Median (IQR)	Min	Max	Median (IQR)	Min	Max
1	Let’s know our bones!	100.0 (80.0)	83.0	100.0	100.0 (50.0)	0	100.0
2	Osteoporosis	100.0 (0)	-	-	100.0 (37.5)	25.0	100.0
3	Adolescent bone health	100.0 (8.0)	92.0	100.0	100.0 (12.5)	75.0	100.0
4	Calcium	100.0 (0)	-	-	100.0 (0)	-	-
5	Vitamin D	100.0 (0)	-	-	100.0 (0)	-	-
6	Physical activity and exercise for bone strength	100.0 (0)	-	-	100.0 (0)	-	-
7	Seven healthy lifestyles for bone health	100.0 (0)	-	-	100.0 (0)	-	-
	Video	100.0 (4.0)	92.0	100.0	100.0	-	-

## Data Availability

The datasets are available upon request from the corresponding author for interested researchers.

## References

[1] Farr J.N., Khosla S. (2015). Skeletal changes through the lifespan-from growth to senescence. Nat. Rev. Endocrinol..

[2] Chin K.-Y., Ng B.N., Rostam M.K.I., Muhammad Fadzil N.F.D., Raman V., Mohamed Yunus F., Syed Hashim S.A., Ekeuku S.O. (2022). A mini review on osteoporosis: From biology to pharmacological management of bone loss. J. Clin. Med..

[3] Stagi S., Cavalli L., Iurato C., Seminara S., Brandi M.L., de Martino M. (2013). Bone metabolism in children and adolescents: Main characteristics of the determinants of peak bone mass. Clin. Cases Min. Bone Metab..

[4] Demontiero O., Vidal C., Duque G. (2012). Aging and bone loss: New insights for the clinician. Ther. Adv. Musculoskelet. Dis..

[5] Alswat K.A. (2017). Gender disparities in osteoporosis. J. Clin. Med. Res..

[6] Bone Health and Osteoporosis Foundation https://www.bonehealthandosteoporosis.org/preventing-fractures/nutrition-for-bone-health/peak-bone-mass/.

[7] International Osteoporosis Foundation https://www.osteoporosis.foundation/patients/about-osteoporosis.

[8] Julián-Almárcegui C., Gómez-Cabello A., Huybrechts I., González-Agüero A., Kaufman J.M., Casajús J.A., Vicente-Rodríguez G. (2015). Combined effects of interaction between physical activity and nutrition on bone health in children and adolescents: A systematic review. Nutr. Rev..

[9] Cheung C.L., Ang S.B., Chadha M., Chow E.S., Chung Y.S., Hew F.L., Jaisamrarn U., Ng H., Takeuchi Y., Wu C.H. (2018). An updated hip fracture projection in Asia: The Asian Federation of Osteoporosis Societies study. Osteoporos. Sarcopenia.

[10] Zulfarina M.S., Sharif R., Syarifah-Noratiqah S.-B., Sharkawi A.M., Aqilah-SM Z.S.-M., Mokhtar S.-A., Nazrun S.A., Naina-Mohamed I. (2018). Modifiable factors associated with bone health in Malaysian adolescents utilising calcaneus quantitative ultrasound. PLoS ONE.

[11] Flynn A. (2003). The role of dietary calcium in bone health. Proc. Nutr. Soc..

[12] Rizzoli R. (2022). Dairy products and bone health. Aging Clin. Exp. Res..

[13] Min S.K., Oh T., Kim S.H., Cho J., Chung H.Y., Park D.H., Kim C.S. (2019). Position Statement: Exercise guidelines to increase peak bone mass in adolescents. J. Bone Metab..

[14] Kohrt M., Bloomfield S., Little K., Nelson M.E., Yingling V.R. (2004). Physical activity and bone health. Med. Sci. Sports Exerc..

[15] Nilsen O.A., Emaus N., Christoffersen T., Winther A., Evensen E., Thrane G., Furberg A.S., Grimnes G., Ahmed L.A. (2021). The influence of snuff and smoking on bone accretion in late adolescence. The Tromsø study, Fit Futures. Arch. Osteoporos..

[16] Malik P., Gasser R.W., Moncayo R., Kemmler G., Wolfgang Fleischhacker W. (2012). Markers of bone resorption and formation during abstinence in male alcoholic patients. Alcohol. Clin. Exp. Res..

[17] American Academic of Orthopaedic Surgeons https://orthoinfo.aaos.org/en/staying-healthy/healthy-bones-at-every-age/.

[18] Cummings S.R., Black D.M., Nevitt M.C., Browner W., Cauley J., Ensrud K., Genant H.K., Palermo L., Scott J., Vogt T.M. (1993). Bone density at various sites for prediction of hip fractures. The Study of Osteoporotic Fractures Research Group. Lancet.

[19] Sözen T., Özışık L., Başaran N.Ç. (2017). An overview and management of osteoporosis. Eur. J. Rheumatol..

[20] Leboime A., Confavreux C.B., Mehsen N., Paccou J., David C., Roux C. (2010). Osteoporosis and mortality. Jt. Bone Spine.

[21] Pan K., Zhang C., Yao X., Zhu Z. (2020). Association between dietary calcium intake and BMD in children and adolescents. Endocr. Connect..

[22] Beto J.A. (2015). The role of calcium in human aging. Clin. Nutr. Res..

[23] Mithal A., Wahl D.A., Bonjour J.P., Burckhardt P., Dawson-Hughes B., Eisman J.A., El-Hajj Fuleihan G., Josse R.G., Lips P., Morales-Torres J. (2009). Global vitamin D status and determinants of hypovitaminosis D. Osteoporos. Int..

[24] Nguyen Bao K.L., Sandjaja S., Poh B.K., Rojroongwasinkul N., Huu C.N., Sumedi E., Aini J.N., Senaprom S., Deurenberg P., Bragt M. (2018). The consumption of dairy and its association with nutritional status in the South East Asian Nutrition Surveys (SEANUTS). Nutrients.

[25] Guthold R., Stevens G.A., Riley L.M., Bull F.C. (2020). Global trends in insufficient physical activity among adolescents: A pooled analysis of 298 population-based surveys with 1.6 million participants. Lancet Child Adolesc. Health.

[26] Chan C.Y., Mohamed N., Ima-Nirwana S., Chin K.-Y. (2018). A review of knowledge, belief and practice regarding osteoporosis among adolescents and young adults. Int. J. Environ. Res. Public Health.

[27] Schettler A.E., Gustafson E.M. (2004). Osteoporosis prevention starts in adolescence. J. Am. Acad. Nurse Pract..

[28] Yoon S., An S., Noh D.H., Tuan L.T., Lee J. (2021). Effects of health education on adolescents’ non-cognitive skills, life satisfaction and aspirations, and health-related quality of life: A cluster-randomized controlled trial in Vietnam. PLoS ONE.

[29] Abdolalipour S., Mirghafourvand M. (2021). Effect of education on preventive behaviors of osteoporosis in adolescents: A systematic review and meta-analysis. Int. Q. Community Health Educ..

[30] Holland A. (2017). Osteoporosis knowledge translation for young adults: New directions for prevention programs. Health Promot. Chronic Dis. Prev. Can..

[31] Rahim F.R., Suherman D.S., Muttaqiin A. (2020). Exploring the effectiveness of e-book for students on learning material: A literature review. J. Phys. Conf. Ser..

[32] Tosun N. (2014). A study on reading printed books or e-books: Reasons for student-teachers preferences. Turk. Online J. Educ. Technol..

[33] Firdausy B.A., Prasetyo Z.K. (2020). Improving scientific literacy through an interactive e-book: A literature review. J. Phys. Conf. Ser..

[34] Castro M., Pilger D., Fuchs F., Ferreira M. (2007). Development and validity of a method for the evaluation of printed education material. Pharm. Pract..

[35] Shoemaker S., Wolf M., Brach C. (2014). Development of the Patient Education Materials Assessment Tool (PEMAT): A new measure of understandability and actionability for print and audiovisual patient information. Patient Educ. Couns..

[36] Wong S.T., Saddki N., Arifin W.N. (2019). Inter-rater reliability of the Bahasa Malaysia version of patient education materials assessment tool. Med. J. Malays..

[37] Win K.T., Hassan N.M., Bonney A., Iverson D. (2015). Benefits of online health education: Perception from consumers and health professionals. J. Med. Syst..

[38] López-Escribano C., Montesino S., García-Ortega V. (2021). The impact of e-book reading on young children’s emergent literacy skills: An analytical review. Int. J. Environ. Res. Public Health.

[39] American Psychological Association https://www.apa.org/news/press/releases/2018/08/teenagers-read-book.

[40] Holzer J., Korlat S., Haider C., Mayerhofer M., Pelikan E., Schober B., Spiel C., Toumazi T., Salmela-Aro K., Käser U. (2021). Adolescent well-being and learning in times of COVID-19-a multi-country study of basic psychological need satisfaction, learning behavior, and the mediating roles of positive emotion and intrinsic motivation. PLoS ONE.

[41] Beets M.W., Cardinal B.J., Alderman B.L. (2010). Parental social support and the physical activity-related behaviors of youth: A review. Health Educ. Behav..

[42] Do U.P., Stenhammar C., Edlund B., Westerling R. (2017). Health communication with parents and teachers and unhealthy behaviours in 15- to 16-year-old Swedes. Health Psychol. Behav. Med..

[43] Hemberg J., Sved E. (2021). The significance of communication and care in one’s mother tongue: Patients’ views. Nord. J. Nurs. Res..

[44] Domínguez M. (2014). Einstein versus Neutrinos: The two cultures revisited with the media coverage of a scientific news item in cartoons. Sci. Commun..

[45] Keçeci A., Toprak S., Kiliç S. (2019). How effective are patient education materials in educating patients?. Clin. Nurs. Res..

[46] Ab Hamid M.R., Mohd Yusof N.D.B., Buhari S.S. (2022). Understandability, actionability and suitability of educational videos on dietary management for hypertension. Health Educ. J..

[47] Jamil N.A., Chau S.H., Abdul Razak N.I., Shamsul Kamar I.I., Mohd-Said S., Rani H., Sameeha M.J. (2021). Development and evaluation of an integrated diabetes-periodontitis nutrition and health education module. BMC Med. Educ..

